# OLDER PEOPLE LIVING WITH DEGENERATIVE SPINE DISEASE WHO UNDERGO SPINE SURGERY—TRYING TO BE NORMAL

**DOI:** 10.1093/geroni/igac059.2454

**Published:** 2022-12-20

**Authors:** Andrea Strayer, Barbara King

**Affiliations:** University of Iowa College of Nursing, Iowa City, Iowa, United States; University of Wisconsin-Madison School of Nursing, Madison, Wisconsin, United States

## Abstract

Lumbar degenerative spine disease (DSD), a consequence of aging, occurs globally in 266 million persons annually. As the population ages, the number of spinal surgeries will increase. The purpose was to investigate older peoples’ understandings of living with and having surgery for DSD and the process they engage in to return to normal. Grounded theory (GT) was used to guide this study. Fourteen older people (≥ 65yrs) were recruited for 2 in-depth interviews (audio-recorded/transcribed verbatim) at 2-time-points: T1 during hospitalization and T2, 1-3-months post-discharge. All 14 interviewed at T1; 10-T2 (1mo); 2-T2 (2mo); 2-T2 (3mo) post-discharge (N=28 interviews). Consistent with GT, purposive and theoretical sampling was used. Data analysis (interdisciplinary team) included open, axial, and selective coding. A conceptual model was developed illustrating the phases older persons with DSD go through in their trajectory of trying to return to normal. Three key categories were identified (1) Losing Me (2) Fixing Me and (3) Recovering Me. All described a prolonged process of losing functional independence and being able to socialize. Fixing Me was proving they needed surgery and preparing for surgery. Recovering Me involved monitoring and ongoing progress. Conditions, including setbacks and delays, slowed recovery. Throughout, participants had to continually adjust their expectations. The conceptual model details how older people engage in living with and undergoing DSD surgery. Our model can serve as the foundation for developing interventions to guide older patient education programs, improve care transitions and develop patient-centered approaches for treating older people with DSD and spine surgery.

